# Brain connectivity in individuals with migraine resets during the headache phase: a whole-brain connectivity study

**DOI:** 10.1093/braincomms/fcaf045

**Published:** 2025-01-30

**Authors:** Enrico Schulz, Astrid Mayr, Pauline Jahn, Anne Stankewitz

**Affiliations:** Department of Radiology, University Hospital LMU, Ludwig-Maximilians-Universität München, Munich 81377, Germany; Department of Medical Psychology, Ludwig-Maximilians-Universität München, Munich 81377, Germany; Department of Radiology, University Hospital LMU, Ludwig-Maximilians-Universität München, Munich 81377, Germany; Department of Neurology, University Hospital LMU, Ludwig-Maximilians-Universität München, Munich 81377, Germany; Department of Diagnostic and Interventional Neuroradiology, School of Medicine, Klinikum rechts der Isar, Technical University of Munich, Munich 81675, Germany

**Keywords:** migraine, functional connectivity, migraine cycle

## Abstract

Episodic migraine is reflected by cyclic changes in behavior and cortical processing. We aimed to identify how functional connectivity changes over the entire migraine cycle. By using longitudinal neuroimaging and a whole-brain connectivity analysis approach, we tested 12 episodic migraine patients across 82 functional MRI recordings during spontaneous migraine headaches with follow-up measurements over the pain-free interval without any external stimulation. We found that the functional connectivity linearly increased over the interictal interval. In the prodromal phase, we observed the strongest connections between the anterior agranular insula and the posterior orbitofrontal cortex with sensory, motor and cingulate areas. The strength of the connections dropped during the headache. Peak connectivity during the prodromal phase and its collapse during the headache can be regarded as a mechanism of normalizing cortical processing. We speculate about a malfunction at the molecular level in agranular frontal and insular regions, which needs to be addressed in subsequent studies.

## Teaser

In a longitudinal study of 12 migraineurs with 82 functional MRI recordings over the migraine cycle, Schulz *et al*. observed that prodromal peak connectivity between the anterior insula and the orbitofrontal cortex with sensory and cingulate regions collapsed during the headache attack—possibly a protective mechanism to prevent sensory overload.

## Graphical Abstract

**graphical abstract:**
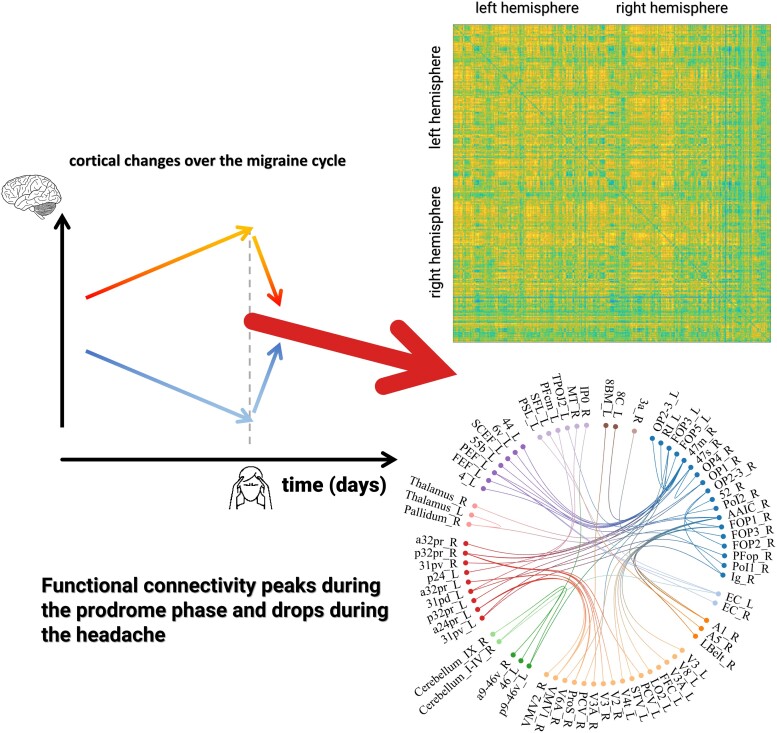

## Introduction

Episodic migraine is a cyclic disease with recurring headache episodes, which are often the most disabling symptom. However, migraine attacks are more than just a headache.^1^ Migraine attacks often begin and end up to 48 h prior to or after the headache with characteristic symptoms.^2,3^ Within this period, a migraine headache typically lasts between 4 and 72 h (=headache phase).^4^ Typical premonitory symptoms during the prodrome phase are e.g. mood changes, muscle stiffness, irritability and fatigue^5^ whilst postdrome symptoms include e.g. tiredness, polyuria and concentration deficits.^6^ However, symptoms of both phases are partially overlapping and some patients report postdromes already during the headache phase.^7^ In one-third of migraine patients, an aura lasting about 5–60 min occurs prior to or during the headache (=aura phase).^4^ Whilst the beginning and the end of headache and aura phases can be relatively easily defined by the patients, it is much more difficult for the prodrome and postdrome phases.^7^

### The cyclic nature of migraine

Evidence for cyclic changes in the brain of individuals with migraine originates from early neurophysiological studies: EEG results revealed a lack of habituation to repetitive sensory stimulation^8^ during the pain-free interval that normalized just before or during the headache phase.^9^ Migraine-phase-dependent brain activity and functional connectivity (FC) have also been observed in functional MRI studies.^10-13^ The most consistent findings of these studies are changes in the hypothalamus, spinal trigeminal nuclei, limbic system and pons during the prodrome and/or headache phases (details on hypothalamic findings are provided in the following paragraph). The involvement of additional brain regions, some functionally related to the primary areas, has also been implicated in migraine pathophysiology.^14,15^

### Prodrome phase

Within the prodrome phase, increased BOLD activity was found in the trigeminal nuclei^16^ and in the hypothalamus^11^ in response to trigeminal pain. Additionally, using a similar pain-inducing model, stronger connections were observed between these two regions.^3^ Evidence from others supports altered limbic processes specifically during the prodrome phase: they found increased limbic activity e.g. in the nucleus accumbens, the insula and the hippocampus,^12^ and also increased connectivity strengths within the limbic circuit^13^ and between the limbic system and the hypothalamus.^12^ Furthermore, whilst Schulte *et al*.^13^ found stronger prodromal connections between the nucleus accumbens and the dorsal rostral pons compared to interictally, Karsan *et al*.^15^ observed that the limbic-pontine FC changed from positive to negative during the nitroglycerine-induced premonitory phase.

### Headache phase

During the headache phase, studies observed stronger ponto-hypothalamic^13^ and ponto-sensory connections to the somatotopic head and face area of the primary somatosensory cortex,^14^ whilst others observed no changes in pontine activity^16,17^ or connectivity.^18^ Ictally, Amin *et al*.^18^ observed increased thalamo-cortical connections, such as to the contralateral orbitofrontal and insular cortices, and decreased thalamo-cortical connections to the ipsilateral somatosensory cortices. Our group further identified a decoupling of hypothalamo-limbic connections and disconnections within thalamic, limbic and sensory functional networks during the headache phase.^19^

### Hypothalamus as a rhythmic generator of migraine attacks

The cyclicity of episodic attacks in individuals with migraine has specifically been attributed to the hypothalamus due to its role in the regulation of biological rhythms and in maintaining internal homeostasis by controlling the endocrine and autonomic nervous systems.^1,5^ Several migraine symptoms and trigger factors of attacks are closely related to the hypothalamus.^7,12^ Consistent with the clinical picture of the disease, neuroimaging studies have provided compelling evidence that the hypothalamus plays a critical role in the initiation and maintenance of migraine attacks.^11-13,20,21^ The first observation of bilateral hypothalamic hyperperfusion during spontaneous headache phases was made with PET.^22^ Subsequent imaging studies did not find hypothalamic changes during headache but during the prodrome.^11-13,20^ Specifically, increased hypothalamic activity during the prodrome phase of spontaneous migraine headache in response to trigeminal pain^11^ and during the nitroglycerine-induced premonitory phase.^20^ In addition, increased connectivity between the hypothalamus and the spinal trigeminal nuclei^13^ and between the hypothalamus and the limbic system^12,13^ was detected during the prodromal phase. In contrast, during the headache phase, stronger connectivity was found between the hypothalamus and the dorsal pons, a region previously discussed as a potential ‘migraine generator’.^13,23^ Our group further found that the connectivity strength between the hypothalamus and limbic regions (e.g. nucleus accumbens, insula and hippocampus) progressively increases during the interictal period, peaking just before headache onset, and then dissociates during the headache phase itself.^12,17^ It is worth noting, however, that some studies, including two previous functional MRI studies using trigemino-nociceptive and olfactory stimulation, have failed to detect hypothalamic changes either during headache phases^16,17^ or within the 72 h prior to spontaneous headache onset.^16^

### The importance of the prodrome phase

Previous research,^9-12,20^ as well as clinical features^7,24^ of the disease (e.g. attack trigger and premonitory symptoms), suggests that the initiation of headache phases most likely starts during the interictal pain free and specifically during the prodrome phase, hours or even days before the headache occurs. However, most imaging studies on migraine have used cross-sectional designs; longitudinal within-subject studies taking the entire migraine cycle and whole-brain activity into account are less common.^10-12,25,26^

Building on our previously published work focusing on resting-state networks and brain perfusion over the migraine cycle,^12,19^ we extend our knowledge of cyclic mechanisms of the brains of individuals with migraine by exploring the trajectory of whole-brain FC during the resting-state functional MRI over the entire migraine cycle. *First*, based on the clinical picture of the disease^5^ and previous imaging findings,^11,12,22^ we hypothesized that there are hypothalamic alterations in connectivity throughout the migraine cycle. *Second*, due to reported electrophysiological ‘dys-responsiveness’ in migraine patients,^8,9,27,28^ we hypothesized that there are cyclic alterations in functional connections involving sensory brain areas.

## Materials and methods

### Ethics approval and consent to participate

All patients gave their written, informed consent. The study was conducted according to the Declaration of Helsinki and approved by the Ethics Committee of the Technische Universität München (project number 90/14).

### Subjects

**Figure 1:**
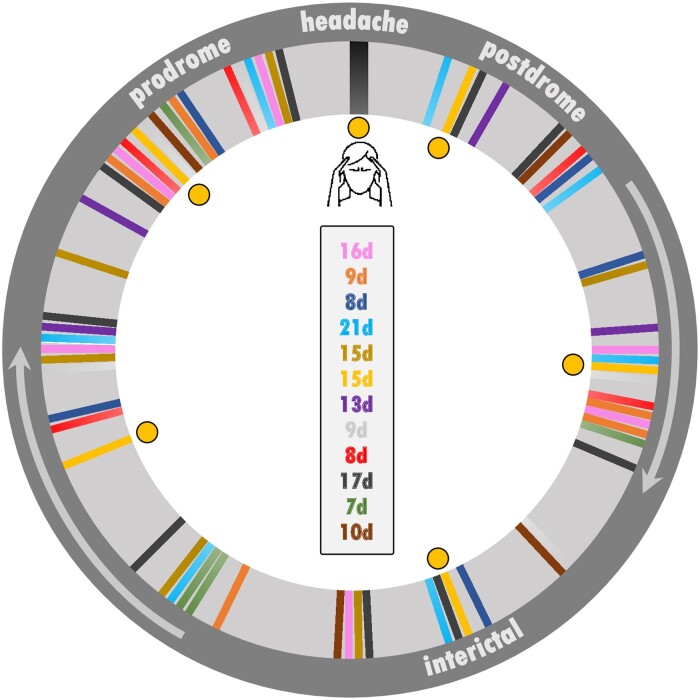
**Circular time course of individual recordings between two migraine attacks.** The figure integrates the acquisition days into a circular representation of a migraine cycle with its different phases: the headache phase at 12 o’clock, the following postdrome, interictal and prodrome phases along the circle. The colored lines indicate all the recordings of the 12 patients (*n* = 82 functional MRI measurements). Each color belongs to the same patient (12 different colors), and the number of lines indicates the number of recordings. Each time series starts with an attack recording (black line at 12 o’clock). The number of days between the first recorded headache phase and the subsequent headache phase (both at 12 o’clock) is shown in the middle and indicates the different lengths of each patient’s migraine cycle as measured in the study. We recorded one migraine cycle per patient. For example, in Patient 1 (=yellow color, highlighted by the yellow circles), the length of the measured migraine cycle was 15 days (see top position in the middle). During these days, the patient was measured once during the headache phase and five times during the pain-free migraine interval (=yellow lines). For other, potentially more comprehensive descriptions of the recording procedure, see our previous publications.^12,19^

Twelve migraine patients were included in the study. Characteristics and clinical features are presented in Table 1. Migraine diagnosis was based on the classification of the International Headache Society.^4^ Patients did not report any other neurological or psychiatric disorders, were not on preventive migraine medication for at least 6 months, but were allowed to take their usual acute migraine medication (nonsteroidal anti-inflammatory drugs or triptans) after the headache phase was recorded. In order to collect a sufficient amount of individual data for our longitudinal statistical analyses, we included patients with attack frequencies between 1 and 6 per month. One patient reported a frequency of between 6 and 10 attacks per month; this patient was also included but tested daily. Figure 1 shows the time course of the functional MRI recordings for the 12 subjects (11 females and 1 male). All patients gave their written, informed consent. The study was conducted according to the Declaration of Helsinki and approved by the Ethics Committee of the Technische Universität München (project number 90/14). All patients were reimbursed 300 euros.

**Table 1:** Demographic characteristics and clinical migraine features of the measured and following headache

Patient	Age (years)	Female (f)/male (m)	Attacks per month	Disease duration (years)	Measured headache						Following headache					
					Attack severity (0–10)	Location of headache	With visual aura	Photophobia	Phonophobia	Nausea (N)/vomiting (V)	Attack severity (0–10)	Location of headache	With visual aura	Photophobia	Phonophobia	Nausea (N)/vomiting (V)
1	28	f	3–6	6	6	Right-sided	Yes	Yes	Yes	Yes (N)	6	Right-sided	Yes	Yes	Yes	Yes (N)
2	24	m	3–6	14	5–6	Right-sided	No	Yes	Yes	No	5	Right-sided	No	Yes	Yes	No
3	26	f	3–6	24	6	Left-sided	No	Yes	No	No	6	Right-sided	No	Yes	No	No
4	40	f	1–2	29	7–8	Right-sided	Yes	Yes	Yes	Yes (N)	6	Right-sided	Yes	Yes	Yes	Yes (N)
5	32	f	1–2	15	6–7	Right-sided	No	Yes	Yes	No	6	Right-sided	No	No	Yes	No
6	30	f	1–2	11	7	Bilateral	No	Yes	No	No	7	Bilateral	No	Yes	No	No
7	22	f	1–2	6	5	Left-sided	No	Yes	Yes	No	6	Left-sided	No	Yes	No	No
8	23	f	3–6	7	8	Bilateral	No	Yes	Yes	Yes (N)	7	Right-sided	No	Yes	Yes	Yes (N)
9	33	f	6–10	23	8	Right-sided	No	Yes	No	Yes (N)	8	Right-sided	No	Yes	Yes	Yes (N, V)
10	26	f	1–2	7	5–7	Left-sided	No	Yes	No	No	6	Left-sided	No	Yes	No	No
11	21	f	3–6	7	8	Bilateral	Yes	Yes	Yes	No	7	Bilateral	Yes	Yes	Yes	No
12	30	f	1–2	14	7	Bilateral	No	Yes	Yes	Yes (N)	7	Bilateral	Yes	Yes	Yes	Yes (N)

Attack severity was recorded on a numerical rating scale ranging from 0 (no pain) to 10 (highest imaginable pain).

This analysis is a secondary analysis of previously collected data. All patients have been previously reported.^12,19^ These prior articles mainly dealt with cortical perfusion and intrinsic network activity. Here, we are utilizing a different aspect and report how the whole-brain connectivity across 408 parcellated brain regions develops over the migraine cycle. No statistical power calculation was conducted prior to the study. The sample size was based on the available data and the feasibility of data collection, particularly regarding migraine phase recordings.

### Study design

Migraine patients underwent repeated testing throughout an entire migraine cycle (see Fig. 1). The functional MRI time series for each patient began with the recording of a spontaneous and untreated headache phase. On the attack day, the resting-state data were recorded within the first 6 h after the beginning of the acute headache phase. Subsequent functional MRI data were then recorded every 1–4 days until patients informed us by telephone about the following headache phase (which was not scanned). The time series was completed with the last attack-free recording. The functional MRI data were recorded for 5–10 days, depending on the cycle length of each patient (see Fig. 1 for the length of each migraine cycle and the distribution of recording days along the cycle). For example, Patient 1 (16d cycle) has been recorded on Days 2, 6, 9, 11, 13 and 15 after the headache phase. We obtained data for all participants (12/12) on the first day after the attack and for the majority of patients (9/12) on the day before or the same day before the subsequent attack. All subjects had their last recording within 48 h before the subsequent attack.

### Image acquisition

MRI data were acquired in a 3 Tesla scanner (Philips Ingenia, The Netherlands) using a 32-channel head coil. Each patient underwent two sequences of ∼10 min duration at each measurement time point in the following order: (i) an arterial spin labelling sequence and (ii) a resting-state functional MRI sequence. Patients were instructed to remain awake and relaxed with their eyes closed. In the present study, the 300 resting-state volumes were analysed using the following parameters: TR = 2000 ms; time to echo (TE) = 30 ms; FOV = 192 × 192 mm^2^; flip angle = 90°; number of slices = 37; voxel size = 3 × 3 × 3 mm^3^ (0.29 mm gap). A high-resolution T1-weighted anatomical image was acquired for image registration: TR = 9000 ms, TE = 4 ms, flip angle = 8°, FOV = 240 × 240 × 170 mm^3^; number of slices = 170; voxel size = 1.0 × 1.0 × 1.0 mm^3^). Field maps were acquired in each session to control for B0 effects; 64 slices, TR = 960 ms, FOV = 192 × 192 mm^2^; voxel size = 2.0 × 2.0 × 2.0 mm^3^, 0.2 mm gap between slices; TE = 6 ms/10.55 ms, flip angle 60°.

### Image pre-processing

MRI data were collected on a 3 Tesla scanner (Ingenia, Philips, The Netherlands) using a 32-channel head coil^19^ and were pre-processed with FSL.^19,29^ Independent component analyses were used to remove large artefacts from each session. On average, 43 ± 4 components were evaluated, and a total of 17 ± 3 artefact components (39 ± 7%) were removed. The time series of functional volumes were projected to surface space by using the ‘Connectome Workbench’ package. Regions of interest (ROIs) were defined by subdividing the cortical surface into 180 regions per hemisphere, plus 11 of the subcortical regions.^30^ We further added 37 regions, including the cerebellum and subcortical regions, such as the periaqueductal grey (PAG), the thalamus, cerebellar subregions and the amygdala. The cerebellar and further subcortical regions were derived from the FSL atlas. The remaining ROIs were created based on the literature, e.g. the PAG. The time courses for all voxels of cortical activity for a specific region of the Glasser Atlas, e.g. the middle insula, were extracted. Nuisance variables (McFLIRT motion parameters, their squares and temporal derivatives with squares; a total of 24 regressors), as well as outliers, were regressed out from the data. Outliers in the functional MRI data are defined by the framewise displacement (FD) and DVARS. A volume is defined as an outlier if it exceeds one of the following thresholds: FD ≧ 0.2 mm or DVARS ≧ (the 75th percentile + 1.5 times the interquartile range). Outliers are marked for each subject and then included in the regression as a vector of zeros (non-outlier) and ones (outlier). The total number of 8.8 ± 5.7 volumes (out of 300) per session was marked; the maximum number of outlier volumes in one session was 29 (9.7%). We computed principal component analyses separately for each ROI and subject and selected the first component (Version R2018a, Mathworks, USA). Time courses of the 408 components were correlated using Kendall’s *τ* coefficient.

### Statistical analyses

We explored the cyclic change of cortical resting-state connectivity between the 408 regions over the migraine interval. To investigate how the cortical connectivities evolve over the trajectory of measurements, we computed linear mixed-effects (LME) models for each pair of brain regions and related the time points within the migraine cycle to the strength of connectivity quantified by Kendall’s *τ*.

**Figure 2:**
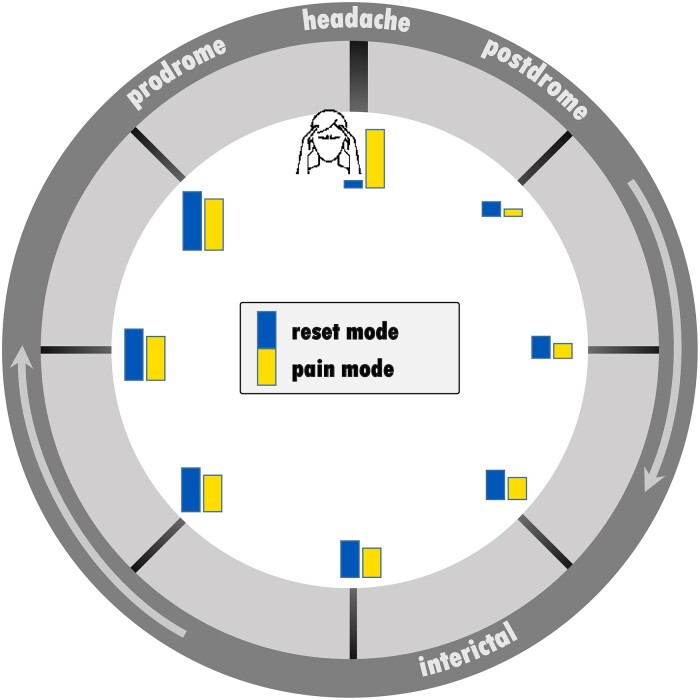
**Circular time series of migraine-related brain processes.** We hypothesized two hypothetical time series (=trajectories) of brain processes (connectivity strength) over the migraine cycle, which we modelled in the statistical analysis. The figure shows these two trajectories in a circular plot representing the migraine cycle with the headache phase at 12 o’clock. In Trajectory 1 (‘reset mode’ = blue bars), connectivity strength increases over the pain-free interval towards the next attack, with peak connectivity during the prodrome (highest blue bar plot) and minimal connectivity during the headache (lowest blue bar plot). In Trajectory 2 (‘pain mode’ = yellow bars), brain processes also increase over the pain-free interval towards the next attack, but peak during the headache (highest yellow bar plot) and then decrease towards the postdrome phase.

We created a time vector for each patient’s migraine cycle and encoded the day of recording by assigning numbers between 1 and 2 to normalize for the different cycle lengths of each patient. The numbers 1 and 2 were assigned to the measurement during the headache phase, depending on the following two different trajectories of connectivity strengths (Fig. 2) in the brain.^12^

Trajectory 1 (‘reset mode’) represents a linear trajectory (increase or decrease) towards the attack with peak connectivity during the prodrome phase, followed by connectivity ‘reset’ to the baseline level during the headache (‘reset mode’). In this model, FC on the day of the headache is similar to that on the day after the headache. For example, for five measurements over 10 days, the following vector is used to represent trajectory one: 1 (=attack), 1.2, 1.4, 1.6, 1.8.

Trajectory 2 (‘pain mode’) exhibits a linear trajectory (increase or decrease) towards the attack with peak connectivity during the headache (‘pain mode’). In this model, FC on the day of the headache phase is similar to that on the day before the headache. Similar to the example above with five measurements over 10 days, the following vector is used for trajectory two: 1.2, 1.4, 1.6, 1.8, 2 (=attack).

To explore the relationship between the fluctuating map strength and the dependence of the time point of the migraine cycle, we computed LMEs that related the longitudinal recordings of resting-state connectivity to the number vector of recording days:


f_connectivity∼cycle+(1|subject)

T-values are computed voxel-wise as quotients between the beta estimates and the standard errors of the equation. The statistical threshold (two-tailed, *P* < 0.05, equivalent to *t* > 5.195) was determined using the ‘palm_datapval.m’ function publicly available in PALM.^31^ This approach corrects for multiple testing. Connectivity figures were generated using NeuroMArVL (https://immersive.erc.monash.edu/neuromarvl/).

## Results

We analysed how the resting-state FC evolves depending on the time point within the migraine cycle. All 66 significant pairs of connectivity for Trajectory 1 have a positive mean across all 82 Kendall’s *τ* values, and 94% of these pairs (*n* = 62) show a higher mean than standard deviation, indicating that the effects are predominantly based on a stronger co-variation of the time courses between two brain regions in the prodrome phase compared to a declined connectivity during the headache phase as reflected by Kendall’s *τ* coefficients around 0 (see Supplementary Table 1 for detailed statistics).

**Figure 3:**
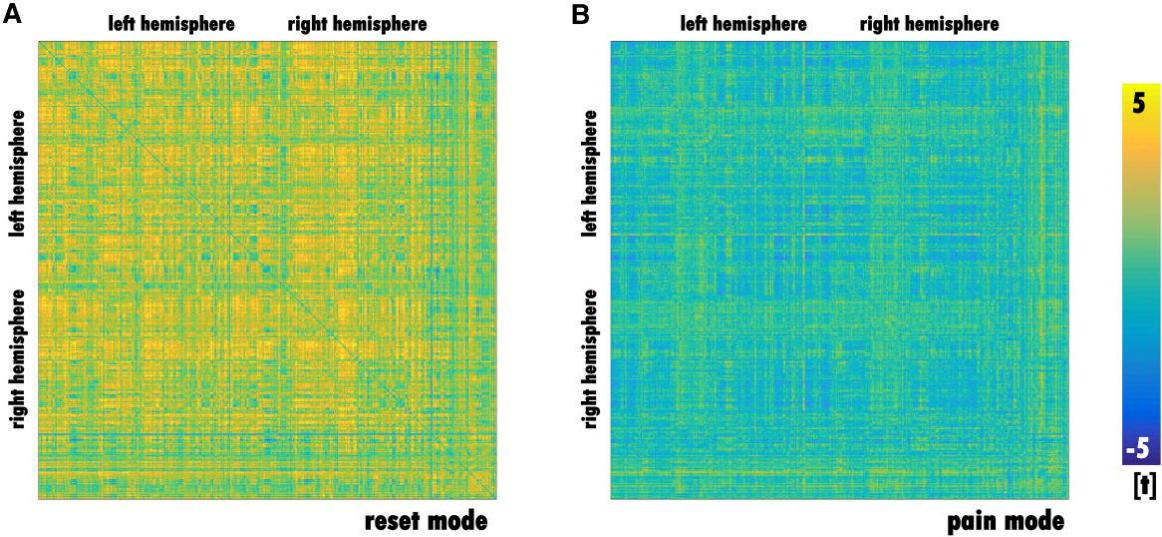
**Confusion matrix on migraine cycle-related connectivity changes across all 408 brain regions.** The matrices show the full results of the LME models based on 12 participants and 82 recordings. Each of the 408 × 408 data points corresponds to a statistical test of the change in connectivity over the migraine cycle for two brain regions. The left matrix (**A**) shows the statistics for Trajectory 1 (reset mode), which peaks at a pain-free time point just before the headache phase. All of these trajectories show an increase in connectivity over time with a drop during the headache phase. There was no decreasing connectivity towards a minimum at the time point just before the attack. The right matrix (**B**) has no significant change in connectivity with the highest connectivity during the headache phase (pain mode).

**Figure 4:**
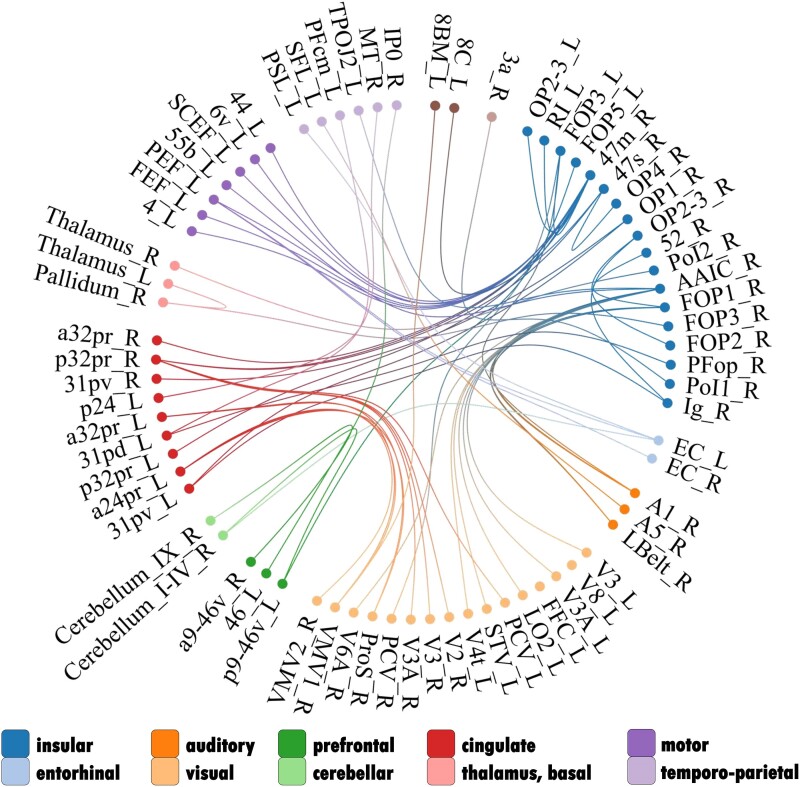
**Cycle-related connectivity changes following the ‘reset mode’ trajectory.** The figure shows a circular plot representing all significant connections based on the LME models, which included 12 participants and 82 recordings (https://immersive.erc.monash.edu/neuromarvl/?save=2ad2c963-1181-47bb-907d-40a47d31e1d1_95.91.248.128). Each line corresponds to a statistical test of the change in connectivity over the migraine cycle for two brain regions. Supplementary Table 2 reports the exact statistical values and defines the abbreviations.

**Figure 5:**
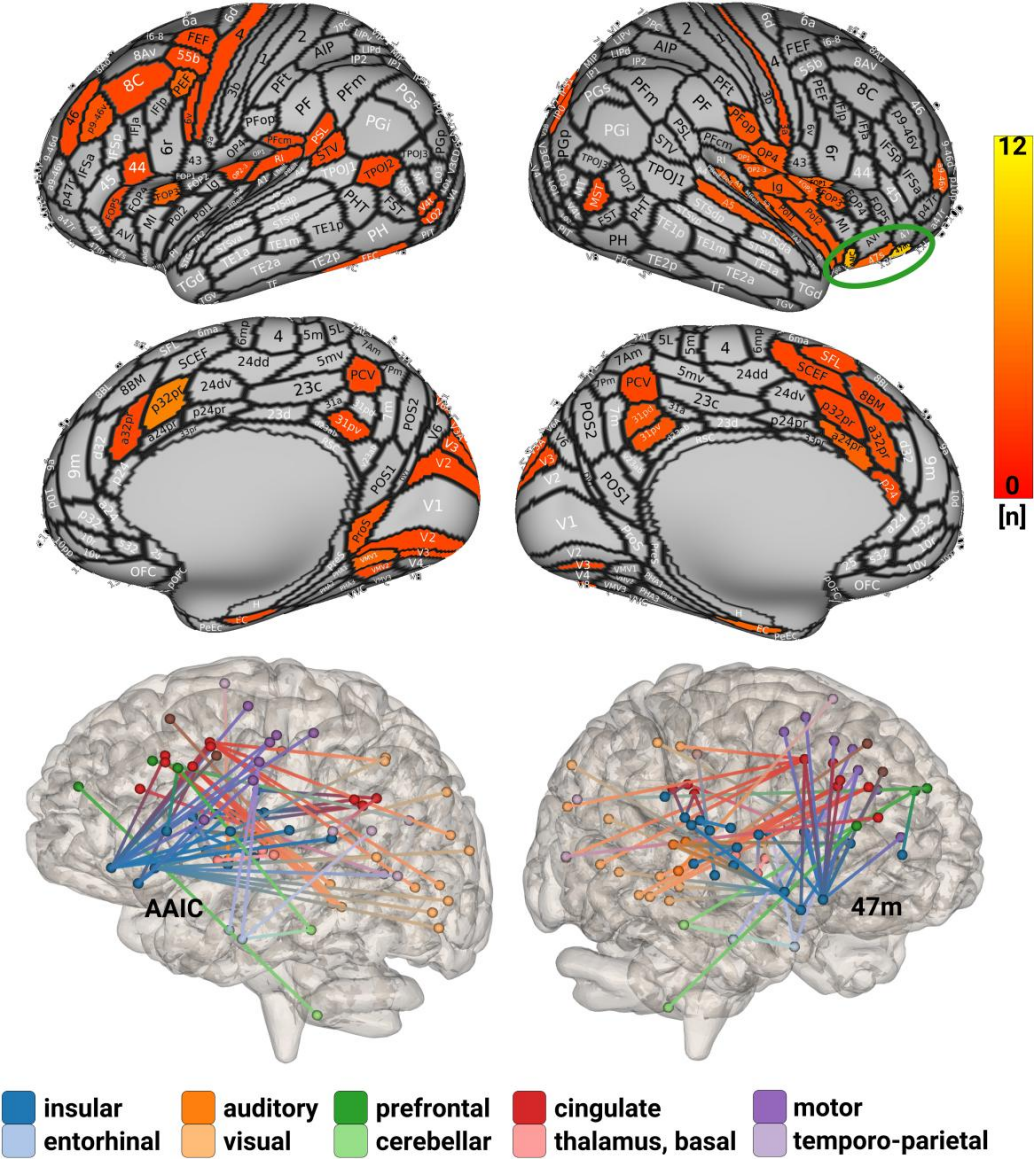
**Cycle-related connectivity changes following the ‘reset mode’ trajectory.** The figure shows the connected brain regions, based on the LME models, which included 12 participants and 82 recordings (https://immersive.erc.monash.edu/neuromarvl/?save=2ad2c963-1181-47bb-907d-40a47d31e1d1_95.91.248.128). The color coding in the upper part indicates the number of connections with the highest number for the area 47 m (12) and the aAIC (10). Supplementary Table 2 reports the exact statistical values and defines the abbreviations.

We have provided three different types of figures to illustrate the same results. A confusion matrix gives a more global impression of the direction of connectivity changes throughout the migraine cycle (Fig. 3). We examined all possible functional connections across the migraine cycle; the *x*-axis and *y*-axis represent the 408 regions. Each square in the confusion matrix represents a statistical test that reflects the change in connectivity across the migraine cycle according to the hypothesized trajectory. Note that our relatively rich confusion matrix can only provide a global view of the effects, not detailed information for each connection. A circular plot shows all significant connections (Fig. 4), and the best-connected brain regions are presented on a 3D brain (Fig. 5). All figures show different aspects of the same underlying results. Two possible time courses were explored:

‘Trajectory 1’: We found four larger complexes of brain regions that account for most of the significant connections: (i) agranular inferior frontal cortex (area 47 m), (ii) agranular insula [anterior agranular insular cortex (aAIC)], (iii) posterior cingulate cortex (PCC; Brodmann area 31) and (iv) anterior cingulate cortex (ACC; Brodmann areas 24 and 32).

- ‘Agranular inferior frontal cortex’: The most affected region is the area ‘47 m’ (=12 connections), which reached its peak/trough prior to the beginning of the headache and drops its connection during the headache phase to insular and parietal opercular regions, the primary somatosensory cortex, regions that process eye movements and the dorsal PCC.
- ‘Agranular insula’: The aAIC (=10 connections) exhibits a steady increase of connectivity prior to the attack and shows a drop of connectivity predominantly to areas related to sensory functions (auditory—lateral belt region, primary auditory cortex; visual—V3, V3A, V4t, V6A; visual integration areas—V6A, TPOJ, superior temporal visual area).
- ‘PCC’: We found several connections between the PCC (BA 31) and the insular-opercular regions.
- ‘ACC’: The dorsal ACC showed multiple connections to the inferior frontal regions (BA 47), visual (V1–V3) and higher-order visual areas [prostriate and ventromedial visual areas (VMVs) 1 and 2].

The complete table of results can be found in Supplementary Table 2.

‘Trajectory 2’: There are no significant effects for this model.

## Discussion

The present longitudinal functional MRI study aimed to detect changes in whole-brain FC in individuals with episodic migraine over the migraine cycle. Based on the clinical picture of the disease^5^ and previous imaging findings,^11,12,22^ we specifically hypothesized cyclic alterations of brain connections in which (i) the hypothalamus and (ii) sensory circuits are involved. By taking the time point of the attack into account, we analysed two possible connectivity trajectories with peak connectivity strengths: (i) during the prodromal phase (trajectory 1 = ‘reset mode’) and (ii) during the headache phase (trajectory 2 = ‘pain mode’). Significant results were found only for the ‘reset mode’.

Contrary to our initial ‘first hypothesis’, we did not identify any significant hypothalamic connections following these two trajectories. This negative result is surprising given that previous data, including our own, have demonstrated hypothalamic alterations in individuals with migraine.^11,12,20^ A possible explanation may be that the present data are the first to be generated longitudinally and then analysed in a whole-brain approach over the entire migraine cycle. In contrast, previous studies have focused exclusively on predefined regions, including the hypothalamus. This approach of analysing and correcting only ‘ROI’ increases the likelihood of exceeding statistical thresholds.^12,13^ Another interpretation could be that previous findings on hypothalamic changes—including our own—were not statistically robust enough for the relatively moderate sample size of the study. It is worth noting that hypothalamic changes have been observed in some, but not all, imaging studies.^16^ Those who observed such hypothalamic dysfunction reported it in different phases of migraine and most of them in response to external pain stimulation and not during natural, experimentally uninfluenced brain activity.^13,20^ This aspect highlights the importance of testing the reliability of study results in general.

Our ‘second hypothesis’ was confirmed by our data showing that the connectivity strength of different sensory brain regions follows the ‘reset mode’ with the strongest connectivity before the headache phase, followed by an uncoupling during the headache. Our results are discussed in detail below.

### Trajectory of the ‘reset mode’

The results suggest that alterations in brain processes develop early on and can be observed and potentially treated before the headache phase is initiated. This is in line with previous neurophysiological research that has identified deviant sensory processing in the interictal phase: studies reported a lack of habituation to sensory stimulation during the interictal phase of the migraine cycle followed by a ‘normalization’ of habituation just before and during the headache phase.^9,32^ These findings served as the theoretical basis for the generation of the vector, where we also modelled a linear increase towards the following attack with a peak during the prodrome and a drop during the headache phase. For this trajectory, we identified three major regions that may represent these deviations.

#### A potential dysfunction of agranular insula and inferior frontal regions

The best-connected region is the right area 47 m, which is located in the inferior frontal cortex. Involvement of this region in the context of migraine has been found in the seminal work of Denuelle *et al.*^22^ Using PET during headache phases—amongst other regions—the Brodmann area 47 was active during the headache as well as after the successful treatment of the headache with analgesic medication, suggesting that this region is involved in the underlying migraine-related cortical processes. In the present study, the area 47 m exhibits a migraine-related cyclic trajectory predominantly with regions in the left insula and with the bilateral posterior part of the ACC (BA 32), which are both known to be involved in the processing of pain^33^ partly due to the increased but unspecific salience of the perceived pain.^34^ Specifically, our data showed peak connectivity between area 47 m and the insula and ACC during the prodrome phase and lowest connectivity during the headache.

Furthermore, we observed various connections in subregions of the bilateral insular cortex affected throughout the migraine cycle. The best-connected insular region is the agranular aAIC. Similar to the above-discussed trajectory of area 47 m, these connections exhibit a steady increase of connectivity strength towards the following headache phase and show an ictal drop, particularly to sensory areas (auditory, higher-order visual). The aAIC has been previously related to the processing and integration of sensory information^35^ as well as part of the salience network.^36^ The anterior insula is also involved in the cognitive aspects of processing pain^37^ and in maintaining working memory functions in mice^38^ and men.^39^ In migraine patients, previous studies found altered FC of the anterior insula to the cerebellum.^40^ In addition, increased anterior insula activity has been observed specifically during the—pharmacologically induced—prodrome phase.^21^

Taken together, the strong involvement of the regions aAIC and area 47 m in sensory and working memory processes suggests the current view on migraine as an overflow of sensory input that cannot be sufficiently emptied from cortical loops that maintain conscious processes. This is in line with neurophysiological findings that interictal migraine patients suffer from the inability to habituate to sensory stimulation in the interictal phase.^8,9^ Similar to the connectivity trajectory of the aAIC and area 47 with the highest connectivity strength immediately prior to the headache and drop to baseline during the headache, habituation in individuals with migraine has been shown to reset to normal during and throughout a headache phase.^9^ Future research is needed to address this hypothesis by directly probing salience and habituation throughout the migraine cycle. Interestingly, the anterior insula and the posterior part of the orbitofrontal cortex are similar regarding gross morphology and architecture^41^; they lack a granular layer 4 and are characterized by a substantial number of von Economo neurons (VENs).^42^ In their original work, von Economo and Koskinas^43^ stated that VENs can be found in ‘cap, corners and interior wall of the frontal part of the Gyrus limbicus, in the posterior transitional gyrus of the orbital part of the frontal lobe (which runs to the anterior frontal orbital Insula), in the so-called Gyrus transversus insulae and its associated Gyri breves accessorii (anteriores) insulae (translated by H.L. Seldon: http://self.gutenberg.org/GetPdfFile.aspx?&bid=3468625, Account required)’. A dysfunction of VENs is associated with increased self-awareness, autonomic control and complex cognitive functions.^42,44,45^ In migraine, the prodrome and ictal migraine phases are typically associated with neurocognitive and vegetative symptoms. Migraine phases are most likely associated with increased mental introspection.^35,46-48^ Further research is needed to investigate the possible relationship between VENs and migraine by examining VEN activity in relation to behavioral measures.

We may see a functional distinction in the migraine-related impairments of sensory processing (i.e. the lack of habituation). Whilst visual and auditory processes might be directly bound to the anterior insula, the area 47 m also exhibits connections to the posterior part of the ACC, which in turn connects with higher-order visual areas. This suggests that the influence of area 47 m on sensory processing is rather indirect and requires the mediation of other cortical regions (see https://immersive.erc.monash.edu/neuromarvl/?save=2ad2c963-1181-47bb-907d-40a47d31e1d1_95.91.248.128).

#### Cingulate connectivities

There are further cycle-related changes in connectivity between the posterior part of the ACC and the higher-order VMVs. Subregions along the ACC have been associated with salient sensory stimulation.^34^ The finding may represent the overly salient processing of sensory information^34^ throughout the migraine cycle due to the lack of habituation.^8^

#### Reset of FC during a headache phase

We and others have previously argued that the underlying cause of migraine is an impairment in processing the overflow of sensory information.^9,12,32^ The overflow through the sensory stream that could not be contained^12^ would make sensory information more salient, preventing habituation, and may explain the current findings of involvement of several bilateral insular subregions and regions in the posterior part of the ACC. A final decoupling of the connection between these regions may contribute either to the initiation of the headache phase in migraine or to an active ‘reset’ of cortical processes during the headache phase. However, in this study, we cannot disentangle the low connectivity due to the ‘reset’ from the low connectivity due to the experience of long-lasting pain, as shown in our previous work.^49,50^ Future studies should address this point by comparing repeated recordings during the headache phase—once during the headache and once immediately after triptan-induced pain relief.

### Circularity of migraine-related effects—potential limitations

#### Migraine cycles

One presupposition of our study is that we consider the migraine-phase-related processes as circular. A clock metaphor may underline our assumptions on circularity; we tested our patients at 12 o’clock (the headache phase) and ended our day-by-day recording series at 11 o’clock (the prodrome phase), which is just before the next headache phase at 12 o’clock. We assume that a recording at 2 o’clock or any other ‘time’ would ‘not’ be different across subsequent clock cycles or migraine cycles. Therefore, an attack recording (at 12 o’clock) should be representative of all attack recordings. Our analysis should not be considered linear but cyclic, which always restarts at 12 o’clock. This is the basis of interpretation of all studies on episodic migraine that compare the attack phase with a time point in the interictal phase. Although previous studies on interictal processes in migraine do not test circularity directly, the underlying assumption is the same, which is a differential effect of ‘time’ during the cycle on cortical processing. In line with other neuroimaging studies, this research considers data from only one recording as representative of all previous and future cortical states of the individual. Therefore, we can assume that a single recording during the headache phase is representative of all headache phases experienced by an individual with migraine.

#### Trajectories

The present study examined two time courses of brain connectivity during the migraine cycle, focusing on predefined shapes. We hypothesized a linear progression towards the next headache phase (or the day before). For example, Trajectory 1 shows the strongest connections immediately before an impending migraine headache, followed by a decoupling and lowest connections during the headache itself. This trajectory may reflect the mechanisms of a ‘reset’ of neuronal activity or connectivity during a headache due to sensory overload during the interictal, pain-free period. This phenomenon has been discussed previously in the migraine literature.^51,52^ However, it is important to acknowledge that other trajectories through the migraine cycle—such as sigmoidal, quadratic or nonlinear patterns—are also possible. In particular, it is possible that the best data fit would be obtained by modelling the period around the headache phase (prodrome, aura, headache and postdrome) using a higher sampling rate during these days, whilst the interictal phase could be relatively neglected. Furthermore, it should be noted that different brain regions may follow distinct trajectories, and it is currently unclear whether identical changes in brain connectivity occur during each migraine cycle or whether there are intra-individual differences between different migraine cycles. These aspects should be specifically addressed in future studies investigating several migraine cycles with a higher number of recordings per patient.

#### Control group

For the current design, we deliberately chose not to include a control group. A control condition is not an end in itself. Instead, we need to control for trajectories that occur by chance and for confounding conditions that co-occur with the migraine cycle but are unrelated to the underlying physiological deviation in individuals with migraine. These confounders are currently unknown but need to be recognized and defined.

We believe we have implemented a modern randomization technique to control for chance by using the subject’s own data. Any time series data from other individuals or patients cannot be used as a control because, by definition, they have no time series effect. Any other unrelated and therefore confounding time series effects would have introduced unwanted and spurious variance into the comparison. Thus, only an appropriate and well-defined control condition eliminates potential confounders. Similarly, a repeated measure of healthy subjects should have a null effect. Another inappropriate example would be to use time series from treated individuals with migraine that include a ‘hidden’ attack phase. These patients would still have the full prodrome course, and by using treated patients as controls, we would eliminate the dynamics of the interictal phase towards the prodrome (see further topics in Supplementary material).

## Conclusion

Connectivity changes exhibited a linear increase over the pain-free interval with a peak prior to the headache and a ‘drop’ during the headache (‘reset mode’). Increasing FC towards the next attack with a peak during the prodrome phase is suggested to reflect the alterations of sensory processing shown in previous behavioral and electrophysiological studies. Consequently, novel treatments using CGRP receptor antagonists administered during the prodrome phase were effective in reducing moderate to severe headaches.^53,54^ The collapse of connectivity during the headache can be regarded as a mechanism of resetting the cortical processing. Depending on the affected connection, the peak of synchronicity of functional connections during the end of the ictal phase of the migraine cycle may contribute to various symptoms during the headache phase of migraine attacks, such as headache, hypersensitivity to sensory modalities and autonomic symptoms. These altered processes are hypothesized to be related to VENs in agranular anterior insular and inferior frontal brain regions. Future studies using more appropriate molecular techniques are needed to follow up on this hypothesis.

## Supplementary Material

fcaf045_Supplementary_Data

## Data Availability

Raw data were generated at the Technical University of Munich. The authors confirm that the data supporting the results of this study are available in the article. Requests for additional data are available from the corresponding authors upon reasonable request. We did not develop code for this study.

## References

[1] Ferrari MD , Goadsby PJ, Burstein R, et al Migraine. Nat Rev Dis Primers. 2022;8(1):2. 35027572 10.1038/s41572-021-00328-4

[2] Giffin NJ , Lipton RB, Silberstein SD, Olesen J, Goadsby PJ. The migraine postdrome: An electronic diary study. Neurology. 2016;87(3):309–313. 27335112 10.1212/WNL.0000000000002789 PMC4955275

[3] Giffin NJ , Ruggiero L, Lipton RB, et al Premonitory symptoms in migraine: An electronic diary study. Neurology. 2003;60(6):935–940. 12654956 10.1212/01.wnl.0000052998.58526.a9

[4] Headache Classification Committee of the International Headache Society (IHS) . Headache classification committee of the international headache society (IHS) the international classification of headache disorders, 3rd edition. Cephalalgia. 2018;38(1):1–211. 10.1177/0333102417738202 29368949

[5] Karsan N , Goadsby PJ. Biological insights from the premonitory symptoms of migraine. Nat Rev Neurol. 2018;14(12):699–710. 30448858 10.1038/s41582-018-0098-4

[6] Bose P , Goadsby PJ. The migraine postdrome. Curr Opin Neurol. 2016;29(3):299–301. 26886356 10.1097/WCO.0000000000000310

[7] Ashina M . Migraine. N Engl J Med. 2020;383(19):1866–1876. 33211930 10.1056/NEJMra1915327

[8] Coppola G , Di Lorenzo C, Schoenen J, Pierelli F. Habituation and sensitization in primary headaches. J Headache Pain. 2013;14:65. 23899115 10.1186/1129-2377-14-65 PMC3733593

[9] Judit Á , Sándor PS, Schoenen J. Habituation of visual and intensity dependence of auditory evoked cortical potentials tends to normalize just before and during the migraine attack. Cephalalgia. 2000;20(8):714–719. 11167900 10.1111/j.1468-2982.2000.00122.x

[10] Meylakh N , Marciszewski KK, Di Pietro F, Macefield VG, Macey PM, Henderson LA. Brainstem functional oscillations across the migraine cycle: A longitudinal investigation. Neuroimage Clin. 2021;30:102630. 33770547 10.1016/j.nicl.2021.102630 PMC8024773

[11] Schulte LH , Mehnert J, May A. Longitudinal neuroimaging over 30 days: Temporal characteristics of migraine. Ann Neurol. 2020;87(4):646–651. 32031707 10.1002/ana.25697

[12] Stankewitz A , Keidel L, Rehm M, et al Migraine attacks as a result of hypothalamic loss of control. Neuroimage Clin. 2021;32:102784. 34425551 10.1016/j.nicl.2021.102784 PMC8379646

[13] Schulte LH , Menz MM, Haaker J, May A. The migraineur’s brain networks: Continuous resting state fMRI over 30 days. Cephalalgia. 2020;40(14):1614–1621. 32830513 10.1177/0333102420951465

[14] Hougaard A , Amin FM, Larsson HBW, Rostrup E, Ashina M. Increased intrinsic brain connectivity between pons and somatosensory cortex during attacks of migraine with aura. Hum Brain Mapp. 2017;38(5):2635–2642. 28240389 10.1002/hbm.23548 PMC6867076

[15] Karsan N , Bose PR, O’Daly O, Zelaya FO, Goadsby PJ. Alterations in functional connectivity during different phases of the triggered migraine attack: Alterations in functional connectivity during triggered migraine attack. Headache. 2020;60(7):1244–1258. 32568433 10.1111/head.13865

[16] Stankewitz A , Aderjan D, Eippert F, May A. Trigeminal nociceptive transmission in migraineurs predicts migraine attacks. J Neurosci. 2011;31(6):1937–1943. 21307231 10.1523/JNEUROSCI.4496-10.2011 PMC6633029

[17] Stankewitz A , May A. Increased limbic and brainstem activity during migraine attacks following olfactory stimulation. Neurology. 2011;77(5):476–482. 21775739 10.1212/WNL.0b013e318227e4a8

[18] Amin FM , Hougaard A, Magon S, et al Altered thalamic connectivity during spontaneous attacks of migraine without aura: A resting-state fMRI study. Cephalalgia. 2018;38(7):1237–1244. 28853611 10.1177/0333102417729113

[19] Stankewitz A , Schulz E. Intrinsic network connectivity reflects the cyclic trajectory of migraine attacks. Neurobiol Pain. 2022;11:100085. 35243179 10.1016/j.ynpai.2022.100085 PMC8861450

[20] Maniyar FH , Sprenger T, Monteith T, Schankin C, Goadsby PJ. Brain activations in the premonitory phase of nitroglycerin-triggered migraine attacks. Brain. 2014;137(Pt 1):232–241. 24277718 10.1093/brain/awt320

[21] Karsan N , Bose RP, O’Daly O, Zelaya F, Goadsby PJ. Regional cerebral perfusion during the premonitory phase of triggered migraine: A double-blind randomized placebo-controlled functional imaging study using pseudo-continuous arterial spin labeling. Headache. 2023;63(6):771–787. 37337681 10.1111/head.14538

[22] Denuelle M , Fabre N, Payoux P, Chollet F, Geraud G. Hypothalamic activation in spontaneous migraine attacks. Headache. 2007;47(10):1418–1426. 18052951 10.1111/j.1526-4610.2007.00776.x

[23] Weiller C , May A, Limmroth V, et al Brain stem activation in spontaneous human migraine attacks. Nat Med. 1995;1(7):658–660. 7585147 10.1038/nm0795-658

[24] Blau JN . Migraine: Theories of pathogenesis. Lancet. 1992;339(8803):1202–1207. 1349944 10.1016/0140-6736(92)91140-4

[25] Schulte LH , May A. The migraine generator revisited: Continuous scanning of the migraine cycle over 30 days and three spontaneous attacks. Brain. 2016;139(Pt 7):1987–1993. 27190019 10.1093/brain/aww097

[26] Marciszewski KK , Meylakh N, Pietro D, et al Fluctuating regional brainstem diffusion imaging measures of microstructure across the migraine cycle. eNeuro. 2019;6(4):ENEURO.0005-0019.2019. 10.1523/ENEURO.0005-19.2019 PMC6658917 31300542

[27] Peng KP , May A. Migraine understood as a sensory threshold disease. Pain. 2019;160(7):1494–1501. 31219950 10.1097/j.pain.0000000000001531

[28] Rahimi MD , Fadardi JS, Saeidi M, Bigdeli I, Kashiri R. Effectiveness of cathodal tDCS of the primary motor or sensory cortex in migraine: A randomized controlled trial. Brain Stimul. 2020;13(3):675–682. 32289696 10.1016/j.brs.2020.02.012

[29] Jenkinson M , Beckmann CF, Behrens TEJ, Woolrich MW, Smith SM. FSL. Neuroimage. 2012;62(2):782–790. 21979382 10.1016/j.neuroimage.2011.09.015

[30] Glasser MF , Coalson TS, Robinson EC, et al A multi-modal parcellation of human cerebral cortex. Nature. 2016;536(7615):171–178. 27437579 10.1038/nature18933 PMC4990127

[31] Winkler AM , Ridgway GR, Webster MA, Smith SM, Nichols TE. Permutation inference for the general linear model. Neuroimage. 2014;92:381–397. 24530839 10.1016/j.neuroimage.2014.01.060 PMC4010955

[32] Kropp P , Gerber WD. Contingent negative variation during migraine attack and interval: Evidence for normalization of slow cortical potentials during the attack. Cephalalgia. 1995;15(2):123–128; discussion 78–79. 7641246 10.1046/j.1468-2982.1995.015002123.x

[33] Tracey I , Mantyh PW. The cerebral signature for pain perception and its modulation. Neuron. 2007;55(3):377–391. 17678852 10.1016/j.neuron.2007.07.012

[34] Liang M , Su Q, Mouraux A, Iannetti GD. Spatial patterns of brain activity preferentially reflecting transient pain and stimulus intensity. Cereb Cortex. 2019;29(5):2211–2227. 30844052 10.1093/cercor/bhz026 PMC6458907

[35] Borsook D , Veggeberg R, Erpelding N, et al The insula: A “hub of activity” in migraine. Neuroscientist. 2016;22(6):632–652. 26290446 10.1177/1073858415601369 PMC5723020

[36] Menon V . Salience network. Brain Mapping. 2015;2:597–611.

[37] Tracey I . Imaging pain. Br J Anaesth. 2008;101(1):32–39. 18556697 10.1093/bja/aen102

[38] Zhu J , Cheng Q, Chen Y, et al Transient delay-period activity of agranular insular cortex controls working memory maintenance in learning novel tasks. Neuron. 2020;105(5):934–946.e5. 32135091 10.1016/j.neuron.2019.12.008

[39] Smith R , Lane RD, Alkozei A, et al Maintaining the feelings of others in working memory is associated with activation of the left anterior insula and left frontal-parietal control network. Soc Cogn Affect Neurosci. 2017;12(5):848–860. 28158779 10.1093/scan/nsx011 PMC5460045

[40] Gollion C , Lerebours F, Nemmi F, et al Insular functional connectivity in migraine with aura. J Headache Pain. 2022;23(1):106. 35982396 10.1186/s10194-022-01473-1 PMC9389744

[41] Öngür D , Ferry AT, Price JL. Architectonic subdivision of the human orbital and medial prefrontal cortex. J Comp Neurol. 2003;460(3):425–449. 12692859 10.1002/cne.10609

[42] Allman JM , Tetreault NA, Hakeem AY, et al The von Economo neurons in the frontoinsular and anterior cingulate cortex. Ann N Y Acad Sci. 2011;1225(1):59–71. 21534993 10.1111/j.1749-6632.2011.06011.x PMC3140770

[43] von Economo C , Koskinas G. Die Cytoarchitektonik der Hirnrinde des erwachsenen Menschen. J. Springer; 1925.

[44] Evrard HC , Forro T, Logothetis NK. Von Economo neurons in the anterior insula of the macaque monkey. Neuron. 2012;74(3):482–489. 22578500 10.1016/j.neuron.2012.03.003

[45] Allman JM , Tetreault NA, Hakeem AY, et al The von Economo neurons in frontoinsular and anterior cingulate cortex in great apes and humans. Brain Struct Funct. 2010;214(5–6):495–517. 20512377 10.1007/s00429-010-0254-0

[46] Gil-Gouveia R , Martins IP. Clinical description of attack-related cognitive symptoms in migraine: A systematic review. Cephalalgia. 2018;38(7):1335–1350. 28847155 10.1177/0333102417728250

[47] Gil-Gouveia R , Oliveira AG, Martins IP. The impact of cognitive symptoms on migraine attack-related disability. Cephalalgia. 2016;36(5):422–430. 26350071 10.1177/0333102415604471

[48] Chiang CC , Starling AJ, Buras MR, Golafshar MA, VanderPluym JH. A pilot exploratory study comparing the King-Devick test (KDT) during and between migraine attacks. Cephalalgia. 2020;40(3):307–312. 31660762 10.1177/0333102419885381

[49] Schulz E , Stankewitz A, Winkler AM, Irving S, Witkovský V, Tracey I. Ultra-high-field imaging reveals increased whole brain connectivity underpins cognitive strategies that attenuate pain. Elife. 2020;9:e55028. 32876049 10.7554/eLife.55028 PMC7498261

[50] Mayr A , Jahn P, Deak B, et al Individually unique dynamics of cortical connectivity reflect the ongoing intensity of chronic pain. Pain. 2022;163(10):1987–1998. 35082250 10.1097/j.pain.0000000000002594

[51] Chen WT , Wang SJ, Fuh JL, Lin CP, Ko YC, Lin YY. Peri-ictal normalization of visual cortex excitability in migraine: An MEG study. Cephalalgia. 2009;29(11):1202–1211. 19558536 10.1111/j.1468-2982.2009.01857.x

[52] Coppola G , Tinelli E, Lepre C, et al Dynamic changes in thalamic microstructure of migraine without aura patients: A diffusion tensor magnetic resonance imaging study. Eur J Neurol. 2014;21(2):287–e213. 24200371 10.1111/ene.12296

[53] Dodick DW , Goadsby PJ, Schwedt TJ, et al Ubrogepant for the treatment of migraine attacks during the prodrome: A phase 3, multicentre, randomised, double-blind, placebo-controlled, crossover trial in the USA. Lancet. 2023;402(10419):2307–2316. 37979595 10.1016/S0140-6736(23)01683-5

[54] Karsan N , Goadsby PJ. Neuroimaging in the pre-ictal or premonitory phase of migraine: A narrative review. J Headache Pain. 2023;24(1):106. 37563570 10.1186/s10194-023-01617-x PMC10416375

