# Supplementary material for: Brain connectivity in individuals with migraine resets during the headache phase: a whole-brain connectivity study
Source: Brain Commun. 2025 Jan 30;7(1):fcaf045. doi: 10.1093/braincomms/fcaf045 (PMC11829205; doi:10.1093/braincomms/fcaf045)
Supplement: fcaf045_Supplementary_Data [file fcaf045_supplementary_data.pdf]

Supplementary Table 1. Migraine cycle-related statistical significant connections for trajectory 1

ROI 1	ROI 2	t-value	mean	std
Lateral Belt Complex	Frontal Opercular Area 1	6.67	0.20	0.07
PreCuneus Visual Area	Frontal Opercular Area 3	6.31	0.33	0.18
Insular Granular Complex	Area OP2-3/VS	6.24	0.22	0.10
Area p32 prime	Third Visual Area	6.03	0.21	-0.01
VentroMedial Visual Area 2	Anterior 24 prime	5.96	0.19	0.06
Area 47m	Area p32 prime	5.95	0.20	-0.01
Auditory 5 Complex	Superior Frontal Language Area	5.87	0.24	0.06
Area 47m	RetroInsular Cortex	5.87	0.21	0.13
cerebellum_24_Vermis_IX	Area anterior 9-46v	5.83	0.21	0.36
Frontal OPercular Area 1	Primary Auditory Cortex	5.81	0.18	0.04
Area 47m	Area p32 prime	5.76	0.33	0.02
Area 47m	Area 8C	5.73	0.23	0.36
Area OP1/SII	Area 31p ventral	5.70	0.21	0.15
Anterior Agranular Insula Complex	Third Visual Area	5.69	0.15	0.07
Entorhinal Cortex	PeriSylvian Language Area	5.69	0.20	0.14
Area 47s	Ventral Area 6	5.68	0.22	0.14
Lateral Belt Complex	Anterior Agranular Insula Complex	5.65	0.17	0.02
Frontal OPercular Area 1	Area 52	5.62	0.17	-0.04
ProStriate Area	Area anterior 32 prime	5.61	0.18	0.05
Area 47m	Premotor Eye Field	5.55	0.26	-0.21
Frontal OPercular Area 2	Area OP2-3/VS	5.54	0.20	0.08
ProStriate Area	Anterior 24 prime	5.51	0.21	0.09
Anterior Agranular Insula Complex	Area V4t	5.50	0.23	0.11
Area Frontal Opercular 5	Area posterior 9-46v	5.49	0.21	0.15
VentroMedial Visual Area 1	Anterior 24 prime	5.48	0.29	0.07
Entorhinal Cortex	Premotor Eye Field	5.45	0.19	0.02
Entorhinal Cortex	Premotor Eye Field	5.45	0.22	-0.09
Area V6A	Anterior Agranular Insula Complex	5.44	0.20	0.10
Area 47m	Frontal OPercular Area 3	5.42	0.21	-0.02
THALAMUS_RIGHT	PosteriorInsular Area2	5.41	0.29	0.16
Area 47m	Supplementary and Cingulate Eye Field	5.37	0.21	-0.03
Area 47m	Frontal Eye Fields	5.35	0.22	0.27
Anterior Agranular Insula Complex	Superior Temporal Visual Area	5.34	0.23	-0.02
Area 47s	Area p32 prime	5.33	0.19	0.16
Area OP4/PV	Area 47s	5.32	0.21	0.08
PALLIDUM_RIGHT	Frontal OPercular Area 3	5.32	0.18	0.27
cerebellum_01_Left_I-IV	Area posterior 9-46v	5.32	0.22	0.01

Anterior Agranular Insula Complex	AreaTemporoParietoOccipital Junction 2	5.31	0.17	0.05
cerebellum_28_Right_X	cerebellum_03_Left_V	5.31	0.14	0.08
AreaPosteriorInsular1	Area 3a	5.31	0.24	0.00
VentroMedial Visual Area 1	Area anterior 32 prime	5.30	0.16	0.19
THALAMUS_LEFT	PALLIDUM_RIGHT	5.28	0.24	-0.11
Insular Granular Complex	Area 31p ventral	5.28	0.23	0.04
Area IntraParietal 0	Area 46	5.27	0.22	0.11
Area 47m	Area OP2-3/VS	5.27	0.17	0.27
Primary Auditory Cortex	Frontal OPercular Area 3	5.26	0.16	-0.02
Area OP1/SII	Area 31p ventral	5.26	0.20	0.11
Anterior Agranular Insula Complex	Area V3A	5.25	0.20	0.21
Anterior Agranular Insula Complex	Area Lateral Occipital2	5.25	0.20	-0.03
Area 31pd	Area PFcm	5.25	0.18	0.14
Area p32 prime	Area V3A	5.24	0.21	0.09
cerebellum_01_Left_I-IV	Entorhinal Cortex	5.24	0.26	-0.08
Area 47m	Area 44	5.23	0.20	0.07
Anterior Agranular Insula Complex	Primary Auditory Cortex	5.23	0.33	0.18
Area p32 prime	Second Visual Area	5.23	0.22	0.10
MiddleTemporalArea	Area posterior 24	5.23	0.21	-0.01
Area 47m	Primary Motor Cortex	5.22	0.19	0.06
Area p32 prime	PreCuneus Visual Area	5.21	0.20	-0.01
VentroMedial Visual Area 1	Area 8BM	5.21	0.24	0.06
Area anterior 32 prime	Area 47m	5.20	0.21	0.13
Anterior Agranular Insula Complex	Area 55b	5.20	0.21	0.36
Area PF opercular	Area 31pd	5.20	0.18	0.04

Supplementary Table 2 | Regions of interest with their centres of gravity

Region	Abbreviations	x	y	z
Primary occipital Cortex	V1	-10	-81	3
Medial Superior Temporal Area	MST	-43	-67	6
Sixth occipital Area	V6	-13	-79	31
Second occipital Area	V2	-10	-80	5
Third occipital Area	V3	-16	-86	6
Fourth occipital Area	V4	-28	-84	-3
Eighth occipital Area	V8	-31	-74	-14
Primary Motor Cortex	4	-28	-19	54
Primary Sensory Cortex	3b	-37	-23	52
Frontal Eye Fields	FEF	-39	-4	50
Premotor Eye Field	PEF	-47	1	39
Area 55b	55b	-47	-1	47
Area V3A	V3A	-15	-89	26

Retro Splenial Complex	RSC	-5	-36	21
Parieto-Occipital Sulcus Area 2	POS2	-9	-70	36
Seventh occipital Area	V7	-23	-82	29
IntraParietal Sulcus Area 1	IPS1	-23	-72	37
Fusiform Face Complex	FFC	-40	-58	-17
Area V3B	V3B	-27	-81	19
Area Lateral Occipital 1	LO1	-38	-85	5
Area Lateral Occipital2	LO2	-43	-81	-4
Posterior Inferotemporal Complex	PIT	-40	-79	-12
Middle Temporal Area	MT	-45	-73	9
Primary Auditory Cortex	A1	-42	-25	11
PeriSylvian Language Area	PSL	-57	-45	25
Superior Frontal Language Area	SFL	-8	15	63
PreCuneus occipital Area	PCV	-6	-51	50
Superior temporal occipital Area	STV	-59	-49	16
Medial Area 7P	7Pm	-6	-66	50
Area 7m	7m	-4	-62	35
Parieto-Occipital Sulcus Area 1	POS1	-11	-59	14
Area 23d	23d	-2	-21	38
Area ventral 23 a+b	v23ab	-4	-56	20
Area dorsal 23 a+b	d23ab	-3	-39	31
Area 31p ventral	31pv	-8	-47	32
Area 5m	5m	-6	-40	63
Area 5m ventral	5mv	-13	-38	50
Area 23c	23c	-10	-31	43
Area 5L	5L	-13	-43	70
Dorsal Area 24d	24dd	-7	-19	49
Ventral Area 24d	24dv	-8	-3	44
Lateral Area 7A	7AL	-18	-50	65
Supplementary and Cingulate Eye Field	SCEF	-6	4	57
Area 6m anterior	6ma	-15	3	66
Medial Area 7A	7Am	-8	-57	61
Lateral Area 7P	7Pl	-13	-70	53
Area 7PC	7PC	-32	-49	60
Area Lateral IntraParietal ventral	LIPv	-28	-56	54
Ventral IntraParietal Complex	VIP	-21	-61	61
Medial IntraParietal Area	MIP	-22	-64	45
Area 1	1	-46	-25	54
Area 2	2	-36	-34	53
Area 3a	3a	-35	-21	43
Dorsal area 6	6d	-33	-13	64
Area 6mp	6mp	-11	-13	68
Ventral Area 6	6v	-57	3	32
Area Posterior 24 prime	p24pr	-3	-2	39
Area 33 prime	33pr	-3	5	31
Anterior 24 prime	a24pr	-4	18	30

Area p32 prime	p32pr	-8	14	39
Area a24	a24	-5	40	-2
Area dorsal 32	d32	-8	41	24
Area 8BM	8BM	-5	31	45
Area p32	p32	-9	50	1
Area 10r	10r	-7	51	-7
Area 47m	47m	-34	31	-14
Area 8Av	8Av	-35	17	51
Area 8Ad	8Ad	-22	26	45
Area 9 Middle	9m	-6	52	26
Area 8B Lateral	8BL	-10	35	53
Area 9 Posterior	9p	-15	45	40
Area 10d	10d	-10	65	12
Area 8C	8C	-40	16	37
Area 44	44	-51	16	13
Area 45	45	-48	28	6
Area 47l (47 lateral)	47l	-44	30	-9
Area anterior 47r	a47r	-39	47	-11
Rostral Area 6	6r	-51	7	18
Area IFJa	IFJa	-41	11	25
Area IFJp	IFJp	-39	3	30
Area IFSp	IFSp	-44	22	23
Area IFSa	IFSa	-44	33	13
Area posterior 9-46v	p9-46v	-41	29	29
Area 46	46	-35	38	29
Area anterior 9-46v	a9-46v	-36	52	9
Area 9-46d	9-46d	-27	45	24
Area 9 anterior	9a	-17	55	27
Area 10v	10v	-4	53	-16
Area anterior 10p	a10p	-25	59	-4
Polar 10p	10pp	-13	62	-12
Area 11l	11l	-25	48	-13
Area 13l	13l	-23	28	-19
Orbital Frontal Complex	OFC	-11	33	-22
Area 47s	47s	-31	20	-18
Area Lateral IntraParietal dorsal	LIPd	-29	-56	45
Area 6 anterior	6a	-24	-4	55
Inferior 6-8 Transitional Area	i6-8	-29	7	55
Superior 6-8 Transitional Area	s6-8	-20	20	57
Area 43	43	-57	0	9
Area OP4/PV	OP4	-57	-12	15
Area OP1/SII	OP1	-46	-21	17
Area OP2-3/VS	OP2-3	-38	-18	17
Area 52	52	-38	-22	1
RetroInsular Cortex	RI	-39	-34	18
Area PFcm	PFcm	-50	-32	21
PosteriorInsular Area2	PoI2	-39	-2	-2

Area TA2	TA2	-51	1	-5
Frontal OPercular Area 4	FOP4	-40	13	6
Middle Insular Area	MI	-36	10	1
Pirform Cortex	Pir	-30	6	-19
Anterior Ventral Insular Area	AVI	-31	23	-4
Anterior Agranular Insula Complex	AAIC	-33	13	-12
Frontal OPercular Area 1	FOP1	-48	2	4
Frontal OPercular Area 3	FOP3	-36	3	11
Frontal OPercular Area 2	FOP2	-41	-4	13
Area PFt	PFt	-53	-27	37
Anterior IntraParietal Area	AIP	-38	-41	43
Entorhinal Cortex	EC	-21	-14	-27
PreSubiculum	PreS	-18	-32	-10
Hippocampus	H	-28	-25	-15
ProStriate Area	ProS	-18	-52	1
Perirhinal Ectorhinal Cortex	PeEc	-28	-9	-33
Area STGa	STGa	-51	12	-15
ParaBelt Complex	PBelt	-52	-27	9
Auditory 5 Complex	A5	-60	-17	-1
ParaHippocampal Area1	PHA1	-20	-36	-14
ParaHippocampal Area 3	PHA3	-31	-39	-14
Area STSd anterior	STSda	-53	-5	-11
Area STSd posterior	STSdp	-52	-33	0
Area STSv posterior	STSvp	-55	-34	-4
Area TG dorsal	TGd	-39	10	-32
Area TE1 anterior	TE1a	-60	-9	-20
Area TE1 posterior	TE1p	-60	-46	-8
Area TE2 anterior	TE2a	-55	-24	-26
Area TF	TF	-42	-23	-28
Area TE2 posterior	TE2p	-49	-46	-19
Area PHT	PHT	-58	-56	2
Area PH	PH	-46	-62	-8
Area TemporoParietoOccipital Junction 1	TPOJ1	-51	-47	10
AreaTemporoParietoOccipital Junction 2	TPOJ2	-49	-60	11
AreaTemporoParietoOccipitalJuncti on3	TPOJ3	-44	-69	17
Dorsal Transitional occipital Area	DVT	-17	-71	31
Area PGp	PGp	-38	-82	24
Area IntraParietal 2	IP2	-39	-49	43
Area IntraParietal 1	IP1	-30	-68	42
Area IntraParietal 0	IP0	-30	-77	28
Area PF opercular	PFop	-60	-22	25
Area PF Complex	PF	-57	-38	36
Area PFm Complex	PFm	-48	-55	42
Area PGi	PGi	-46	-59	25
Area PGs	PGs	-40	-73	38
Area V6A	V6A	-20	-84	40
VentroMedial occipital Area 1	VMV1	-18	-54	-6

VentroMedial occipital Area 3	VMV3	-27	-60	-9
ParaHippocampal Area 2	PHA2	-29	-36	-13
Area V4t	V4t	-44	-77	1
Area FST	FST	-46	-66	1
Area V3CD	V3CD	-34	-86	12
Area Lateral Occipital 3	LO3	-44	-79	11
VentroMedial occipital Area 2	VMV2	-26	-54	-6
Area 31pd	31pd	-10	-51	36
Area 31a	31a	-5	-40	41
Ventral occipital Complex	VVC	-29	-52	-17
Area 25	25	-4	23	-14
Area s32	s32	-6	35	-13
posterior OFC Complex	pOFC	-12	12	-19
AreaPosteriorInsular1	PoI1	-38	-13	-3
Insular Granular Complex	Ig	-37	-13	13
Area Frontal Opercular 5	FOP5	-35	26	5
Area posterior 10p	p10p	-21	60	7
Area posterior 47r	p47r	-43	43	1
Area TG Ventral	TGv	-43	-3	-42
Medial Belt Complex	MBelt	-45	-16	2
Lateral Belt Complex	LBelt	-42	-30	9
Auditory 4 Complex	A4	-62	-24	8
Area STSv anterior	STSva	-53	-9	-16
Area TE1 Middle	TE1m	-62	-29	-13
Para-Insular Area	PI	-44	-2	-14
Area anterior 32 prime	a32pr	-8	29	29
Area posterior 24	p24	-4	35	17
Primary occipital Cortex	V1	13	-78	4
MedialSuperiorTemporalArea	MST	44	-64	5
Sixth occipital Area	V6	18	-76	32
Second occipital Area	V2	12	-77	7
Third occipital Area	V3	20	-87	10
Fourth occipital Area	V4	30	-81	-1
Eighth occipital Area	V8	32	-71	-13
Primary Motor Cortex	4	31	-17	54
Primary Sensory Cortex	3b	38	-22	52
Frontal Eye Fields	FEF	43	-2	50
Premotor Eye Field	PEF	47	4	35
Area 55b	55b	50	2	44
Area V3A	V3A	19	-86	29
RetroSplenial Complex	RSC	6	-34	23
Parieto-Occipital Sulcus Area 2	POS2	12	-69	37
Seventh occipital Area	V7	28	-80	33
IntraParietal Sulcus Area 1	IPS1	27	-69	38
Fusiform Face Complex	FFC	40	-53	-18
Area V3B	V3B	30	-77	22
Area Lateral Occipital 1	LO1	39	-81	6
Area Lateral Occipital2	LO2	43	-81	-3
Posterior InferoTemporal Complex	PIT	42	-79	-11

MiddleTemporalArea	MT	48	-70	8
Primary Auditory Cortex	A1	44	-20	10
PeriSylvian Language Area	PSL	62	-36	25
Superior Frontal Language Area	SFL	9	12	65
PreCuneus occipital Area	PCV	7	-53	51
Superior Temporal occipital Area	STV	58	-40	17
Medial Area 7P	7Pm	8	-67	52
Area 7m	7m	6	-60	35
Parieto-Occipital Sulcus Area 1	POS1	12	-56	16
Area 23d	23d	4	-20	38
Area ventral 23 a+b	v23ab	5	-51	21
Area dorsal 23 a+b	d23ab	5	-37	33
Area 31p ventral	31pv	9	-46	33
Area 5m	5m	7	-39	66
Area 5m ventral	5mv	12	-41	54
Area 23c	23c	11	-32	43
Area 5L	5L	15	-45	71
Dorsal Area 24d	24dd	7	-19	52
Ventral Area 24d	24dv	10	-6	45
Lateral Area 7A	7AL	21	-52	66
Cingulate Eye Field	SCEF	6	1	59
Area 6m anterior	6ma	18	3	66
Medial Area 7A	7Am	11	-59	61
Lateral Area 7P	7Pl	16	-69	55
Area 7PC	7PC	33	-48	60
Area Lateral IntraParietal ventral	LIPv	30	-55	54
Ventral IntraParietal Complex	VIP	22	-61	60
Medial IntraParietal Area	MIP	25	-64	47
Area 1	1	47	-23	54
Area 2	2	39	-31	51
Area 3a	3a	35	-19	43
Dorsal area 6	6d	36	-12	62
Area 6mp	6mp	16	-12	68
Ventral Area 6	6v	58	5	29
Area Posterior 24 prime	p24pr	5	-1	40
Area 33 prime	33pr	4	8	30
Anterior 24 prime	a24pr	5	18	32
Area p32 prime	p32pr	9	12	40
Area a24	a24	5	38	0
Area dorsal 32	d32	9	38	25
Area 8BM	8BM	6	27	49
Area p32	p32	9	47	0
Area 10r	10r	7	49	-8
Area 47m	47m	33	32	-15
Area 8Av	8Av	37	19	49
Area 8Ad	8Ad	23	27	45
Area 9 Middle	9m	7	52	24
Area 8B Lateral	8BL	11	37	51
Area 9 Posterior	9p	18	49	35

Area 10d	10d	10	65	7
Area 8C	8C	40	17	37
Area 44	44	52	19	11
Area 45	45	51	29	5
Area 47l (47 lateral)	47l	45	32	-11
Area anterior 47r	a47r	38	51	-7
Rostral Area 6	6r	51	10	15
Area IFJa	IFJa	42	17	25
Area IFJp	IFJp	38	8	28
Area IFSp	IFSp	47	27	20
Area IFSa	IFSa	46	37	8
Area posterior 9-46v	p9-46v	44	31	29
Area 46	46	36	38	29
Area anterior 9-46v	a9-46v	38	51	11
Area 9-46d	9-46d	29	48	24
Area 9 anterior	9a	17	60	23
Area 10v	10v	5	53	-15
Area anterior 10p	a10p	24	61	-7
Polar 10p	10pp	13	62	-14
Area 11l	11l	26	47	-14
Area 13l	13l	22	28	-18
Orbital Frontal Complex	OFC	10	34	-23
Area 47s	47s	31	21	-18
Area Lateral IntraParietal dorsal	LIPd	33	-54	47
Area 6 anterior	6a	27	-2	54
Inferior 6-8 Transitional Area	i6-8	34	9	55
Superior 6-8 Transitional Area	s6-8	20	17	59
Area 43	43	57	1	10
Area OP4/PV	OP4	56	-10	15
Area OP1/SII	OP1	44	-20	19
Area OP2-3/VS	OP2-3	38	-17	18
Area 52	52	39	-20	2
RetroInsular Cortex	RI	42	-30	17
Area PFcm	PFcm	49	-27	23
PosteriorInsular Area2	PoI2	40	-2	0
Area TA2	TA2	51	2	-6
Frontal OPercular Area 4	FOP4	39	15	6
Middle Insular Area	MI	38	10	2
Pirform Cortex	Pir	32	7	-19
Anterior Ventral Insular Area	AVI	35	24	-4
Anterior Agranular Insula Complex	AAIC	34	16	-11
Frontal OPercular Area 1	FOP1	47	4	6
Frontal OPercular Area 3	FOP3	36	7	11
Frontal OPercular Area 2	FOP2	41	-2	14
Area PFt	PFt	56	-23	40
Anterior IntraParietal Area	AIP	40	-38	45
Entorhinal Cortex	EC	21	-15	-26
PreSubiculum	PreS	20	-32	-8
Hippocampus	H	29	-22	-16

ProStriate Area	ProS	19	-47	0
Perirhinal Ectorhinal Cortex	PeEc	27	-9	-33
Area STGa	STGa	51	15	-17
ParaBelt Complex	PBelt	57	-16	7
Auditory 5 Complex	A5	60	-14	-2
ParaHippocampal Area1	PHA1	21	-34	-15
ParaHippocampal Area 3	PHA3	33	-37	-15
Area STSd anterior	STSda	52	0	-16
Area STSd posterior	STSdp	49	-25	-4
Area STSv posterior	STSvp	59	-26	-6
Area TG dorsal	TGd	39	13	-32
Area TE1 anterior	TE1a	60	-3	-24
Area TE1 posterior	TE1p	62	-41	-11
Area TE2 anterior	TE2a	54	-16	-30
Area TF	TF	42	-21	-27
Area TE2 posterior	TE2p	49	-42	-18
Area PHT	PHT	59	-51	-2
Area PH	PH	48	-60	-11
Area TemporoParietoOccipital Junction 1	TPOJ1	53	-40	9
AreaTemporoParietoOccipital Junction 2	TPOJ2	54	-56	8
AreaTemporoParietoOccipitalJuncti on3	TPOJ3	44	-64	16
Dorsal Transitional occipital Area	DVT	21	-67	29
Area PGp	PGp	42	-78	25
Area IntraParietal 2	IP2	44	-44	44
Area IntraParietal 1	IP1	36	-64	44
Area IntraParietal 0	IP0	35	-72	30
Area PF opercular	PFop	61	-18	26
Area PF Complex	PF	59	-31	37
Area PFm Complex	PFm	53	-48	41
Area PGi	PGi	49	-57	24
Area PGs	PGs	45	-66	38
Area V6A	V6A	23	-81	44
VentroMedial occipital Area 1	VMV1	17	-53	-7
VentroMedial occipital Area 3	VMV3	29	-57	-9
ParaHippocampal Area 2	PHA2	30	-33	-15
Area V4t	V4t	45	-76	1
Area FST	FST	47	-62	-1
Area V3CD	V3CD	36	-81	15
Area Lateral Occipital 3	LO3	46	-75	11
VentroMedial occipital Area 2	VMV2	28	-51	-7
Area 31pd	31pd	11	-51	38
Area 31a	31a	7	-41	41
Ventral occipital Complex	VVC	30	-45	-18
Area 25	25	4	20	-13
Area s32	s32	5	35	-12
posterior OFC Complex	pOFC	12	13	-19
AreaPosteriorInsular1	PoI1	39	-11	-3

Insular Granular Complex	Ig	38	-13	14
Area Frontal Opercular 5	FOP5	38	27	4
Area posterior 10p	p10p	25	59	6
Area posterior 47r	p47r	45	44	-3
Area TG Ventral	TGv	38	-1	-43
Medial Belt Complex	MBelt	46	-15	4
Lateral Belt Complex	LBelt	48	-23	9
Auditory 4 Complex	A4	64	-17	6
Area STSv anterior	STSva	55	-8	-17
Area TE1 Middle	TE1m	64	-23	-14
Para-Insular Area	PI	44	-2	-12
Area anterior 32 prime	a32pr	9	27	31
Area posterior 24	p24	5	35	16
Accumbens_left	Accumbens_left	-7	9	-8
Accumbens_right	Accumbens_right	8	11	-8
Amygdala_left	Amygdala_left	-23	-5	-20
Amygdala_right	Amygdala_right	24	-4	-20
Brain_stem	Brain_stem	0	-30	-33
Hippocampus_left	Hippocampus_left	-25	-23	-14
Hippocampus_right	Hippocampus_right	26	-22	-14
Pallidum_left	Pallidum_left	-19	-4	-2
Pallidum_right	Pallidum_right	20	-4	-2
Thalamus_left	Thalamus_left	-11	-19	6
Thalamus_right	Thalamus_right	12	-18	6
HypothalamusASL	HypothalamusASL	6	0	-12
HypothalamusSphere	HypothalamusSphere	6	-6	-12
LCbilat	LCbilat	3	-38	-23
Left_Caudate	Left_Caudate	-13	9	10
Left_Putamen	Left_Putamen	-25	0	1
PAG	PAG	1	-34	-8
PonsSphere2	PonsSphere2	4	-20	-20
Right_Caudate	Right_Caudate	14	10	11
Right_Putamen	Right_Putamen	26	2	0
SpinalSphere	SpinalSphere	6	-40	-46
Cerebellum_Left_I-IV	Cerebellum_Left_I-IV	-7	-44	-17
Cerebellum_Right_I-IV	Cerebellum_Right_I-IV	10	-43	-18
Cerebellum_Left_V	Cerebellum_Left_V	-13	-50	-19
Cerebellum_Right_V	Cerebellum_Right_V	14	-51	-19
Cerebellum_Left_VI	Cerebellum_Left_VI	-23	-59	-25
Cerebellum_Vermis_VI	Cerebellum_Vermis_VI	1	-71	-21
Cerebellum_Right_VI	Cerebellum_Right_VI	24	-58	-25
Cerebellum_Left_Crus_I	Cerebellum_Left_Crus_I	-36	-68	-32
Cerebellum_Right_Crus_I	Cerebellum_Right_Crus_I	38	-68	-32
Cerebellum_Left_Crus_II	Cerebellum_Left_Crus_II	-26	-75	-42
Cerebellum_Vermis_Crus_II	Cerebellum_Vermis_Crus_II	0	-75	-31
Cerebellum_Right_Crus_II	Cerebellum_Right_Crus_II	26	-76	-41
Cerebellum_Left_VIIb	Cerebellum_Left_VIIb	-26	-66	-51

Cerebellum_Vermis_VIIb	Cerebellum_Vermis_VIIb	0	-68	-31
Cerebellum_Right_VIIb	Cerebellum_Right_VIIb	28	-65	-50
Cerebellum_Left_VIIIa	Cerebellum_Left_VIIIa	-24	-57	-53
Cerebellum_Vermis_VIIIa	Cerebellum_Vermis_VIII a	0	-67	-38
Cerebellum_Right_VIIIa	Cerebellum_Right_VIIIa	26	-58	-53
Cerebellum_Left_VIIIb	Cerebellum_Left_VIIIb	-17	-50	-55
Cerebellum_Vermis_VIIIb	Cerebellum_Vermis_VIII b	0	-63	-42
Cerebellum_Right_VIIIb	Cerebellum_Right_VIIIb	18	-51	-55
Cerebellum_Left_IX	Cerebellum_Left_IX	-7	-53	-48
Cerebellum_Vermis_IX	Cerebellum_Vermis_IX	0	-56	-37
Cerebellum_Right_IX	Cerebellum_Right_IX	7	-53	-48
Cerebellum_Left_X	Cerebellum_Left_X	-21	-37	-45
Cerebellum_Vermis_X	Cerebellum_Vermis_X	1	-48	-35
Cerebellum_Right_X	Cerebellum_Right_X	22	-37	-46

Challenges of assessing circular processes in episodic migraine

Q: How did the authors adjust the data based in terms of movement inside the scanner, anxiety, depression, BMI, hypertension, physical exercise level, smoking status, alcohol use, sex, headache frequency, number of total scans and analgesics use?

A: There is a plethora of factors that may influence the cortical processing of migraine. Adequate control of the proposed factors would require a much larger number of subjects equally represented for the different levels of the proposed factors (e.g., an equal or comparable number of men and women, a comparable number of subjects for different headache frequency groups, etc.). Each additional explanatory factor must be supported by additional data. To include all these factors in the longitudinal analysis would require hundreds of recordings. Nevertheless, the above results have the potential to answer the basic question posed in the study, which is to investigate the influence of the timing of the migraine cycle on cortical processing. A recent study (Marek et al., 2022) has questioned this type of analysis due to limited sample size.

Q: How does the parcellation work? Did the authors perform a ROI-to-ROI analysis or on the contrary a cluster-wise analysis? Can the data be presented as a 3D brain image?

A: The analysis is basically a multiple region of interest (ROI) analysis. The boundaries of the regions are defined by the Glasser atlas (Glasser et al., 2016). We did not perform statistics on individual voxels, but performed a PCA on the entire ROI and extracted the first component. For this reason, there is no maximum *t* value with a specific MNI coordinate. The individual ROIs were defined by individual 2D surface projections. Therefore, there is no common MNI coordinate.

Q: Why did the authors do a spatial smoothing of 5 mm?

A: We were also interested in subcortical regions; small nuclei often do not exceed 5 mm in diameter.

Q: *Does the randomisation approach to determine the significance threshold also correct for multiple comparisons?*

A: Yes.

Q: The length of the cycle differs and the number of recordings for each subject, too? Is there a way to deal with this?

A: Ideally, we would have obtained the same number of recordings from patients with identical cycle lengths. However, the length of the migraine cycle is truly variable and largely unpredictable. This means that we inevitably had to deal with either different numbers of recordings or variable gaps between recordings. We decided on a reasonable compromise between the two extrema.

When estimating the slope of the regression line, the first and last data points of the cycle have the strongest influence; these are the times of the migraine cycle that we were most interested in. Thus, the post-attack follow-up began the day after the headache attack. For all patients, the last data were collected 48 hours before the next headache attack. 10 of the 12 patients were recorded one day before the next headache attack. For economic reasons, the other interictal recordings were evenly distributed along the cycle with gaps

between 1 day (short cyclers) and 4 days (long cyclers). The statistical model does not require the same number of recordings or the same cycle length. We have collected enough data to fit the regression parameters. See the figure from our previous publication (Stankewitz and Schulz, 2022) on the same data set.

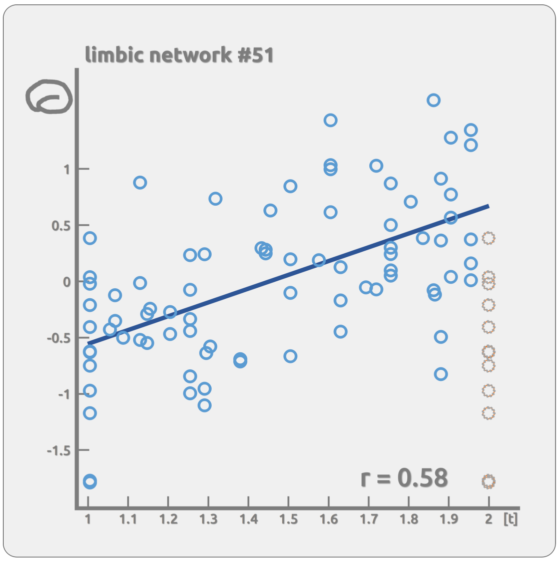

Supplementary Figure 1 | The x-axis shows the normalised time course of the migraine cycle. Time points 1 and 2 represent the headache attack days. The y-axis reflects the changes in cortical processing across the migraine cycle. This example figure is based on data from a previous publication (Stankewitz and Schulz, 2022). The significant correlation ($r=0.58$, $p<0.05$) shows an alternative statistical test ($n=12$, 82 recordings) for the relationship between recording days and cortical processing.

Q: Does the model account for the different number of recordings?

It is important to note that mixed linear models handle unbalanced data very well. Thus, the unbalanced representation of cases with different frequencies of headache is not a problem in terms of the quality of the estimated fixed factors (model parameters). Therefore, a linear mixed effects model is a suitable tool for modelling unbalanced data. In addition, the number of recordings could be included in the model, provided that it has a systematic effect on the results. For example, longer migraine cycles result in a higher number of recordings than shorter cycles. However, the idea that there might be systematic differences in the pathophysiology of short- and long-cycle migraineurs does not seem plausible.

Q: Why do the authors call the time courses "trajectories"?

A: The term "trajectory" is certainly a metaphor used in medicine and developmental science to describe the progress of language or cognitive skills. The term "trajectory" emphasises the determined and cyclical nature of migraine attacks. A simple "time course 1" or "time course 2" would sound too passive and the semantics would suggest processes that just sort of happen. Therefore, in our view, the term trajectory is still best suited to describe *predefined* and ultimately inevitable time courses of neuronal activity.

The shape of the trajectory is predefined, and other shapes are potentially plausible. For the present study, we decided to assume a linear progression towards the next attack (or the day before). However, a non-linear quadratic or other exponential progression might also be plausible. There may be some differences of taste in the use of metaphorical terminology. Metaphorical terminology with temporal connotations such as "evolve," "emerge," and "unfold" clearly helps to make scientific work more digestible.

Q: Why is there no control group included?
A: This is not possible in this study. The reason is that there is nothing comparable to the migraine cycle. For the patients, we coded the migraine cycle with numbers from 1 to 2. For example, if a recording occurred exactly between two attacks, we assigned the number 1.5 to that recording, assuming that the level of brain activity in the middle of the cycle would be halfway between the first and second attack. This principle does not work for a control group, where we would have to assume the same "number" for each recording. The fixed effect of an LME would of course be a null result. This is similar to a correlation between two time series: 1 to 10 correlates with ten times the number 5, for which r=NaN. [in Matlab: correlation = corr((1:10)',5+zeros(10,1))].

Q. How can you assume a change of connectivity for the second headache phase if participants were not scanned during a second migraine?

We interpret our results as circular rather than infinite linear processes. Based on previous neurophysiological research, we assume that the processes we describe in our analysis occur repeatedly in a very similar way. Otherwise, circular processes (e.g. in chronobiology) would always require a certain number of repetitions. This is also true for all migraine studies that specifically examine selected phases of the migraine cycle. Most studies include only one recording per patient and cycle point, assuming that ONE recording of a subject represents ALL possible recordings of that subject.
In line with the current interpretation of neuroimaging findings, we can indeed assume that ONE postdrome recording reflects ALL postdrome recordings. Challenging this assumption would mean that the results of ANY study would require a second or multiple confirmations of the same mental or physical state of a subject. Studies that examine cortical processes during the ictal phase compared to the headache phase are assumed to be valid in the follow-up migraine cycle. Although these studies do not test for circularity, the underlying assumption is the same, namely that one recording represents all possible follow-up recordings.

Q: The imaging time series for each patient started with the recording of a spontaneous, untriggered and untreated headache attack within the first 6 hours after the beginning of the headache. Does this mean that the first scan performed in each patient was during a migraine attack?
A: Yes.

Q: How has the statistical threshold been determined and has the statistics been corrected for multiple testing?
A: Using randomized time vector data, the entire LME analysis was repeated 5000 times, resulting in 5000*83028 statistical tests. The highest absolute t values of each of the 5000 replicates were extracted. This procedure resulted in a right skewed distribution of 5000 values. Based on this distribution, the statistical threshold was determined using the publicly available function "palm_datapval.m" in PALM (Winkler et al., 2016, 2014). A test

was considered "significant" if it exceeded the threshold provided by PALM ($p<0.05$), which corresponds to a t-value of 5.195 for the original test.

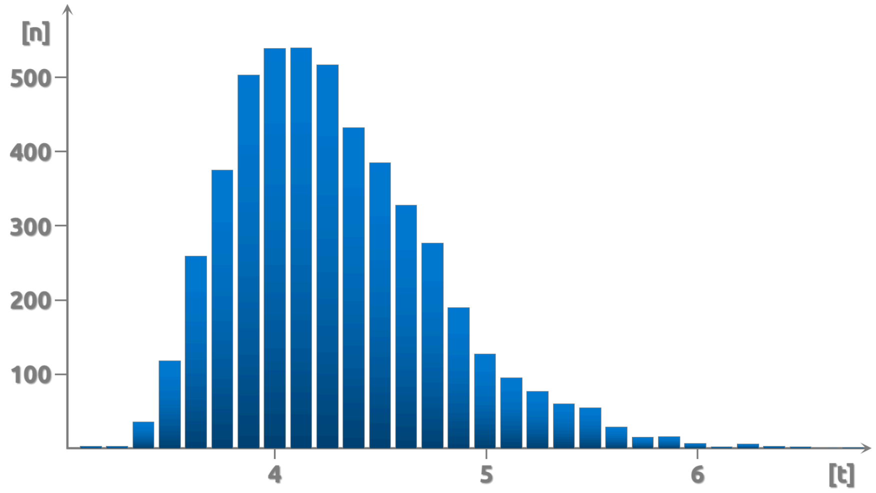

Supplementary Figure 2 | Distribution of statistics on randomised data. The distributions show the absolute maximal values for each of the 5000 randomisations of the linear mixed-effects models (n=12, 82 recordings).

Q: Do our data show a sensory deficit and a lack of habituation?
A: No, they don’t.

Q: Why are the sensory deficit and habituation discussed although we did assess either of them?
A: In the Discussion section, we speculate on how our current findings fit into the literature. Previous studies have already shown that both aspects are impaired in migraineurs compared to healthy controls. Our current findings build on this knowledge. In our view, the discussion section is the appropriate place to interpret our findings. Speculation can stimulate further research and generate hypotheses that can be pursued in subsequent studies.

Q: Have the assumptions required to interpret the statistical analyses been verified?
A:

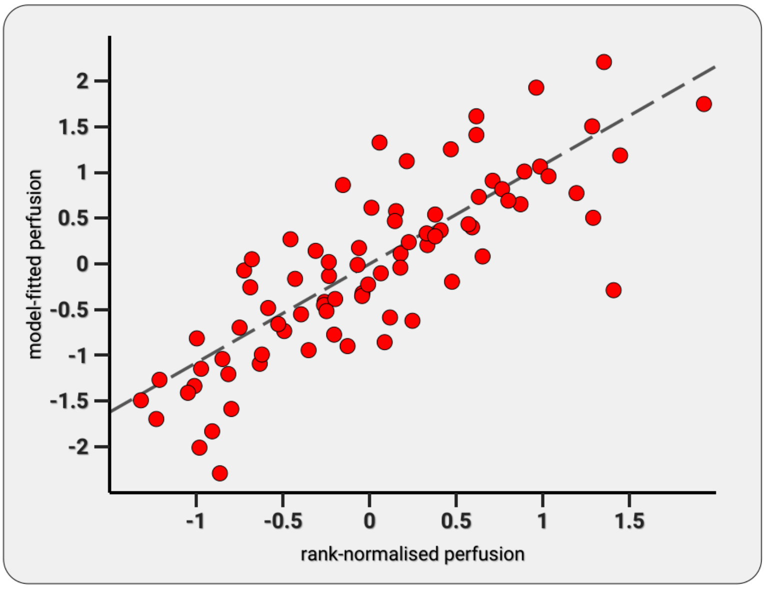

Supplementary Figure 3 | Model fit. For one connection, we tested the fit between the original rank-normalised connectivity data and the fitted connectivity data that were determined by the model parameters (random effects, fixed effect) and the migraine cycle days. The figure shows an excellent model fit (r=0.82, $p<0.05$).

Supplementary References

Glasser MF, Coalson TS, Robinson EC, Hacker CD, Harwell J, Yacoub E, Ugurbil K, Andersson J, Beckmann CF, Jenkinson M, Smith SM, Van Essen DC. 2016. A multi-modal parcellation of human cerebral cortex. *Nature* **536**:171–178. doi:10.1038/nature18933

Marek S, Tervo-Clemmens B, Calabro FJ, Montez DF, Kay BP, Hatoum AS, Donohue MR, Foran W, Miller RL, Hendrickson TJ, Malone SM, Kandala S, Feczko E, Miranda-Dominguez O, Graham AM, Earl EA, Perrone AJ, Cordova M, Doyle O, Moore LA, Conan GM, Uriarte J, Snider K, Lynch BJ, Wilgenbusch JC, Pengo T, Tam A, Chen J, Newbold DJ, Zheng A, Seider NA, Van AN, Metoki A, Chauvin RJ, Laumann TO, Greene DJ, Petersen SE, Garavan H, Thompson WK, Nichols TE, Yeo BTT, Barch DM, Luna B, Fair DA, Dosenbach NUF. 2022. Reproducible brain-wide association studies require thousands of individuals. *Nature*. doi:10.1038/s41586-022-04492-9

Stankewitz A, Schulz E. 2022. Intrinsic network connectivity reflects the cyclic trajectory of migraine attacks. *Neurobiol Pain* **11**:100085. doi:10.1016/j.ynpai.2022.100085

Winkler AM, Ridgway GR, Webster MA, Smith SM, Nichols TE. 2014. Permutation inference for the general linear model. *Neuroimage* **92**:381–397. doi:10.1016/j.neuroimage.2014.01.060

Winkler AM, Webster MA, Brooks JC, Tracey I, Smith SM, Nichols TE. 2016. Non-parametric combination and related permutation tests for neuroimaging: NPC and Related Permutation Tests for Neuroimaging. *Hum Brain Mapp* **37**:1486–1511. doi:10.1002/hbm.23115
